# Gut mutualists can persist in host populations despite low fidelity of vertical transmission

**DOI:** 10.1017/ehs.2022.38

**Published:** 2022-09-02

**Authors:** Xiyan Xiong, Sara L. Loo, Mark M. Tanaka

**Affiliations:** 1 School of Biotechnology and Biomolecular Sciences, University of New South Wales, Sydney, NSW 2052, Australia; 2 Evolution and Ecology Research Centre, University of New South Wales, Sydney, NSW 2052, Australia

**Keywords:** Microbiota, mathematical modelling, host–microbe association, cultural transmission, horizontal transmission

## Abstract

Humans harbour diverse microbial communities, and this interaction has fitness consequences for hosts and symbionts. Understanding the mechanisms that preserve host–symbiont association is an important step in studying co-evolution between humans and their mutualist microbial partners. This association is promoted by vertical transmission, which is known to be imperfect. It is unclear whether host–microbial associations can generally be maintained despite ‘leaky’ vertical transmission. Cultural practices of the host are expected to be important in bacterial transmission as they influence the host's interaction with other individuals and with the environment. There is a need to understand whether and how cultural practices affect host–microbial associations. Here, we develop a mathematical model to identify the conditions under which the mutualist can persist in a population where vertical transmission is imperfect. We show with this model that several factors compensate for imperfect vertical transmission, namely, a selective advantage to the host conferred by the mutualist, horizontal transmission of the mutualist through an environmental reservoir and transmission of a cultural practice that promotes microbial transmission. By making the host–microbe association more likely to persist in the face of leaky vertical transmission, these factors strengthen the association which in turn enables host–mutualist co-evolution.

## Graphical abstract

**Figure:**
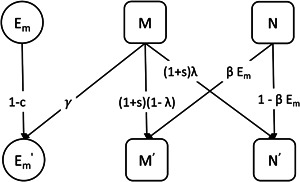

**Social media summary**: Horizontal transmission and human culture help gut mutualists to persist in hosts under imperfect vertical transmission.

## 1. Introduction

Humans with their microbiota are a form of loose symbiosis (Rosenberg & Zilber-Rosenberg, 2018). The gut microbiota is established in individuals through maternal inheritance and through acquisition of microbes from the environment. The microbiota is vertically transmitted from mother to infant through vaginal birth and breast-feeding (Ferretti et al., 2018; Makino et al., 2013; Duranti et al., 2017); however, the transmission is far from perfect and bacterial species sometimes fail to be transmitted to offspring. Furthermore, the adoption of new medical or cultural practices, such as undergoing caesarean section, formula feeding (Blaser, 2017), food fermentation (Kim et al., 2016; Kort et al., 2015) and transportation, can alter the transmission of gut microbes, and have a lasting impact on the structure of the microbiota (Xiong et al., 2021).

The combination of a host (animal or plant) together with its microbiome has been referred to as a holobiont (Margulis et al., 1991), and the collection of genes of the holobiont as the hologenome (Zilber-Rosenberg & Rosenberg, 2008). The holobiont theory suggests that the holobiont is a distinct biological entity during development and in evolution (Margulis et al., 1991; Zilber-Rosenberg & Rosenberg, 2008). Variation of the hologenome includes changes in the microbiome, and plays a fundamental role in the co-evolution between the host and the symbiont as a holobiont (Zilber-Rosenberg & Rosenberg, 2008). The adaptation of the mutualist *Bifidobacteria* to human milk is an example of host–microbe co-evolution (Sela et al., 2008); evidence of cospeciation between Bifidobacteriaceae and the Hominidae has been reported (Moeller et al., 2016). Using mathematical modelling it has been argued that a holobiont that includes mutualists has a selective advantage which leads to a higher abundance of mutualists in the host over time (Roughgarden, 2020).

The holobiont theory is currently a point of contention, however. Opponents of the theory question whether the holobiont is an adequate unit of selection because the selective interests of the host may not align with those of the symbiont (Foster et al., 2017; Stencel & Wloch-Salamon, 2018). Further, it has been argued that the holobiont cannot evolve as a unit since vertical transmission is unstable (Skillings, 2016; Douglas & Werren, 2016). Modelling work has shown that selection at the host level allows beneficial microbes to evolve even when this trait comes at a cost to themselves, although this requires strong vertical transmission (van Vliet & Doebeli, 2019). It remains unclear whether the association between the host and the gut microbiota is strong enough to consider the holobiont as a biological unit.

To study host–mutualist coevolution it is critical to understand the basic mechanisms that preserve or disrupt the association between mutualists and their hosts. In addition to understanding the effects of imperfect vertical transmission from parent to offspring, it is important to study horizontal transmission and the acquisition of microbes from the environment (Rothschild et al., 2018; Blum et al., 2013; Obadia et al., 2017). Gut microbes are found in both free-living and host-associated habitats such as residential homes (Lax et al., 2014; Täubel et al., 2009) and water sources (Fragiadakis et al., 2019). In this paper, we study horizontal transmission through the indirect process of individuals shedding microbes into the environment combined with individuals acquiring microbes from the environment. This mechanism is sensitive to the daily activities of the host, which in turn are greatly affected by the cultural milieu of the host population (David et al., 2014; Pehrsson et al., 2016; Gacesa et al., 2022). As a result, human culture may have a role in tightening the host–mutualist association. Adopting a new cultural practice (such as a dietary practice) can alter the rate of horizontal transmission of a mutualist. For example, the consumption of fermented foods has been shown to promote the establishment of bacterial genera (*Lactobacillus*, *Lactococcus*, *Streptococcus*, *Leuconostoc* and *Bifidobacterium*) that are considered to be mutualists in the gut (Kim et al., 2016; Kort et al., 2015). While cultural practices may affect both horizontal and vertical transmission, we focus here on their effects on horizontal transmission.

Here, we consider whether a mutualist can be maintained in a host population despite unfaithful vertical transmission. In doing so we do not seek to support or critique the holobiont theory; rather we address the more fundamental question about forces that affect the ecological association between microbes and hosts. To this end, we develop and analyse a mathematical model in which a mutualist can be lost between generations owing to leaky vertical transmission, and re-introduced into the population from the environment through horizontal transmission. We consider the effect of cultural factors on the persistence of the mutualist in the population by modelling a cultural practice that affects the horizontal transmission of the mutualist and which is itself transmitted in the host population through social learning. We find that a combination of horizontal microbial transmission and transmitted cultural practices can compensate for the imperfect vertical transmission of the mutualist. This implies that cultural evolution can promote the association and co-evolution between hosts and mutualist symbionts.

## 2. Methods

We construct a deterministic model of a host population with associated microbes that are transmitted through discrete, non-overlapping generations. Hosts reproduce asexually, which can be viewed as a process that tracks female lineages and their associated microbes. In addition to microbes associated with the host population, the model tracks bacteria in the environment. We start with a basic model with a homogeneous host population in order to focus on the effect of imperfect vertical transmission and horizontal transmission on a mutualist in the microbiota. We then extend the model to include cultural factors by adding another type of host; this host engages in a cultural practice that facilitates the horizontal transmission of the mutualist.

### 2.1. Basic model without cultural factors

The model tracks two host–microbiota combinations in the population: one type (*M*^+^) carries the mutualist and the other (*M*^−^) does not. The proportions of *M*^+^ and *M*^−^ in the population are represented by *M* and *N* respectively, and *M* + *N* = 1. The mutualist competes with other bacteria in the environment; the proportions of these bacteria in the environment are *E*_*m*_ and *E*_*o*_ respectively, and *E*_*m*_ + *E*_*o*_ = 1. Since the mutualist, by definition, benefits hosts, individuals with the *M*^+^ microbiota type have a survival advantage, denoted by *s*. We assume, however, that a fitness trade-off applies to mutualists such that their specialised ability to grow well in the host comes with a reduced ability to grow in the environment (Ferenci, 2016). Let *c* be this associated fitness cost in the environment. Individuals with the *M*^+^-type microbiota shed the mutualist into the environment at rate *γ*, which contributes to the proportion of the mutualist in the environment in the next generation, 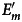.

**Figure 1.**
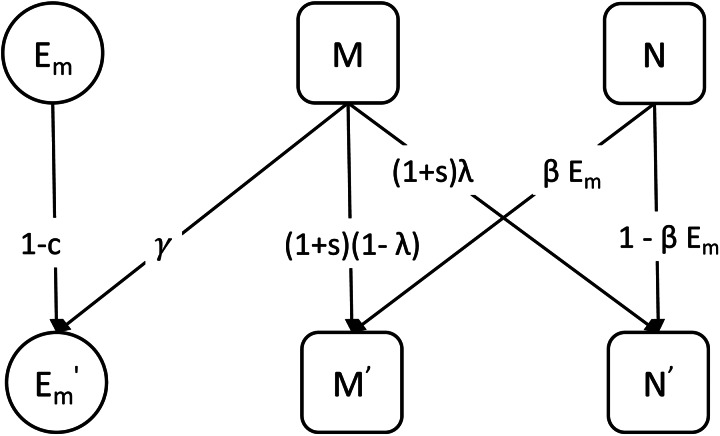
A schematic showing the transmission or change of microbiota types across one generation (indicated by the arrows) in the basic model. *M* is the proportion of hosts that carry the mutualist *M*^+^ and *N* is the proportion of hosts that lack the mutualist *M*^−^. The primes (′) indicate variables in the next generation. The mutualist is shed into the environment with probability *γ* and acquired from the environment with probability *βE*_*m*_. We refer to the combination of these processes as horizontal transmission. The mutualist can fail to transmit to the next generation owing to leaky vertical transmission with probability *λ*.

The probability that a parent fails to transmit the mutualist during reproduction is *λ*, which we describe as leaky vertical transmission. That is, an individual with the *M*^+^ microbiota type can produce an *M*^−^ offspring. The mutualist can be horizontally transmitted indirectly via the environmental population. An individual with the *M*^−^ type microbiota produces *M*^+^ offspring by acquiring the mutualist from the environment with probability *βE*_*m*_. We refer to the combination of shedding and acquisition of the mutualist as horizontal transmission. A schematic of the model is shown in Figure 1.

The model is governed by the following equations.1
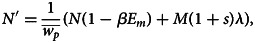
2
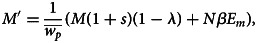
3
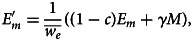
4
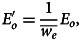
where5
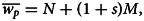
6
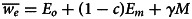
are normalisers to ensure that host variables and environment variables each sum to unity. A summary of model parameters is shown in Table 1.

**Table 1.** Summary of parameters in the model; the top section gives basic model parameters and the bottom section gives extra parameters in the model with cultural factors

Symbol	Description
*γ*	Rate of the mutualist being shed into the environment
*λ*	Rate of imperfect vertical transmission
*β*	Rate parameter for acquiring the mutualist from the environment
*c*	Fitness cost to the mutualist in the environment
*s*	Fitness advantage to host carrying the mutualist (*M*^+^)
*α*	Transmission boost in host *Y* acquiring the mutualist from environment
*k*	Rate constant for host type *X* converting to *Y* through cultural transmission
*δ*	Rate of host type *Y* abandoning the cultural practice

### 2.2. Model with cultural factors

**Figure 2.**
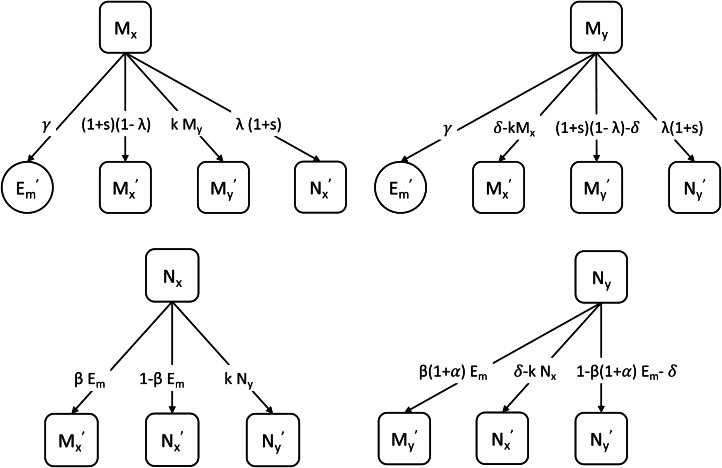
Schematic of the model with cultural factors. The parameters are defined in Table 1. The arrows indicate the transmission or loss of microbes or the cultural practice across one generation. The primes (′) indicate variables in the next generation.

The basic model above does not consider heterogeneity in the way hosts interact with microbes in the environment. In reality, the horizontal transmission and acquisition of microbiota is highly variable between hosts owing to factors such as diet. To study the effect of different cultural practices on the persistence of the mutualist in the gut, we introduce two types of host (*X* and *Y*) in an extended version of the model. Hosts of type *Y* engage in a cultural practice that increases the transmission of the mutualist by a factor *α* compared with hosts of type *X* who do not engage in the cultural practice of the mutualist (Figure 2). The model has four host–microbiota combinations: *M*_*y*_ represents hosts with the mutualist and the cultural practice of interest; *N*_*y*_ represents hosts who have adopted the cultural practice but lack the mutualist; *M*_*x*_ represents hosts with the mutualist but not the cultural practice; and *N*_*x*_ represents hosts lacking the mutualist and the cultural practice. The environmental microbe variables *E*_*m*_ and *E*_*o*_ are as defined previously.

The cultural practice is itself transmitted when hosts interact. A fraction *kM*_*y*_ or *kN*_*y*_ of the offspring of host type *X* adopts the cultural practice in each new generation through interaction with host type *Y*. In this way, a parent without the cultural practice (*X*) can produce offspring with the cultural practice (*Y*). This model therefore includes oblique cultural transmission from all members of the parental generation to offspring (Cavalli-Sforza & Feldman, 1981). The cultural practice can be lost between generations as a fraction *δ* of the offspring of host type *Y* abandons the practice (Figure 2). The offspring of an *N*_*x*_ (or *N*_*y*_) parent can acquire the mutualist from the environment and become an *M*_*x*_ (or *M*_*y*_) individual; an *M*_*x*_ (or *M*_*y*_) parent may fail to transmit the mutualist and thus produce *N*_*x*_ (or *N*_*y*_) offspring (Figure 2).

The dynamics are governed by the following recursions:7
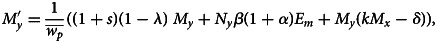
8
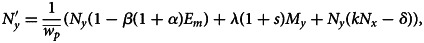
9
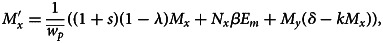
10
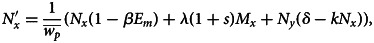
11
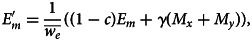
12
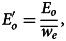
where13
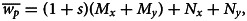
14
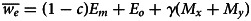
are normalisers to ensure that host variables (*M*_*y*_, *N*_*y*_, *M*_*x*_ and *N*_*x*_) and environment variables (*E*_*m*_ and *E*_*o*_) each sum to unity. A summary of model parameters is shown in Table 1.

## 3. Results

### 3.1. Environmental source and host fitness advantage can compensate for imperfect vertical transmission

To understand the forces that sustain the host–mutualist association, we investigate the conditions under which the mutualist can persist in the population despite imperfect vertical transmission. By doing this we investigate how steps in the transmission process can compensate for deficiencies in other steps. We have derived three steady states of the system (Equations 1–4 and Figure 1) and analysed their stability conditions (Table 2). The stability of the steady states depends on two threshold levels of leakiness in vertical transmission, *λ*. The lower and the upper thresholds are
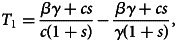
and
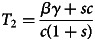
respectively. In equilibrium 1, the other bacteria in the environment that compete with the mutualist are absent (*E*_*o*_ = 0, *E*_*m*_ = 1). In equilibrium 2, both types of microbiota and bacteria in the environment are present (the interior equilibrium). In equilibrium 3, the mutualists from both the population and the environment are absent (*M* = 0, *E*_*m*_ = 0).

**Table 2.** Non-negative equilibria of the basic model and the corresponding conditions for stability

	Equilibrium 1*	Equilibrium 2	Equilibrium 3
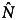	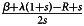	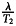	1
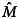	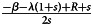	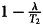	0
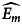	1	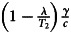	0
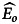	0	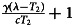	1
Stability conditions	*λ* < *T*_1_	*T*_1_ < *λ* < *T*_2_	*λ* > *T*_2_

*Here we define 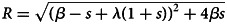.

We summarise the equilibria and their corresponding stability conditions (necessary and sufficient) in Table 2. The full mathematical analysis can be found in the Supplementary Material 1.1.

**Figure 3.**
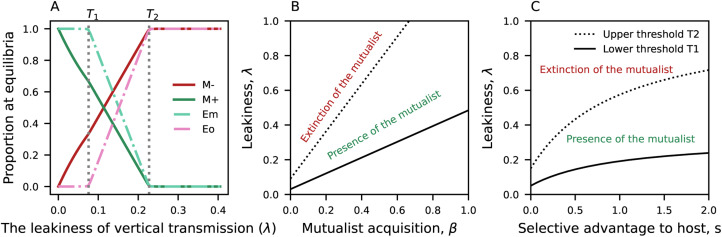
Dynamics of equilibria and threshold values in a culturally homogeneous population (the basic model) obtained by solving Equations (1)–(4). (a) The proportion of microbiota types at equilibria against *λ* (the leakiness of microbial vertical transmission), from numerical solutions. An estimate of the equilibrium is obtained when the difference between two consecutive iterations is smaller than an error of 1 × 10^−5^. The leakiness decreases the proportion of the mutualist in the population *M*^+^ and the environment *E*_*m*_. The black dotted lines represent the lower and upper thresholds (*T*_1_ and *T*_2_), as labelled. (b) and (c) The thresholds of *λ* shift the equilibria of the basic model as a function of *β* and *s* (Table 2). Unless indicated otherwise, the parameters are set at *γ* = 0.15, *β* = 0.1, *s* = 0.1 and *c* = 0.1.

To visualise the relationships among the three equilibria, we plot the steady states of the model as functions of *λ* (Figure 3a). The stable interior equilibrium (equilibrium 2, in which a proportion of the population carries the mutualist) occurs when *λ* lies between the two thresholds *T*_1_ and *T*_2_ (dotted black lines in Figure 3a), and the mutualist is extinct when *λ* is greater than*T*_2_. This is also shown in the longitudinal dynamics (Figure S1 in the Supplementary Material).

We consider the effect of the fitness cost *c* to the mutualist in the environment on the stability of these states by observing that *T*_1_ = *T*_2_(1 − *c*/*γ*). As *T*_2_ is always positive, *T*_1_is always smaller than *T*_2_ (Table 2). The sign of *T*_1_ depends on whether the fitness cost *c* is larger than the rate of microbial shedding by the host, *γ*. If the mutualist shedding (to the environment) is higher than the cost of the mutualist in the environment, i.e. 0 < *c* < *γ*, then *T*_1_ > 0. If 0 < *βγ* < *c* ≤ *γ* then *T*_1_ > 0 and *T*_2_ < 1. Therefore, the condition for a stable interior equilibrium is *T*_1_ < *λ* < *T*_2_. As the value of *λ* increases from 0 to 1 it passes through *T*_1_ and *T*_2_, and the stable equilibrium shifts from equilibrium 1 to equilibrium 2, and finally to equilibrium 3 (extinction of the mutualist), as shown in Figure 3. Sufficiently leaky vertical transmission leads to the extinction of the mutualist in the population and the environment (equilibrium 3). Nevertheless, a degree of leakiness is tolerated for some parameter combinations which allow part of the population to carry the mutualist (equilibria 1 and 2); in Figure 3a where *λ* < *T*_2_, the mutualist is able to persist in the population (solid green line).

Extinction of the mutualist can be prevented if the rates of microbial shedding and acquisition from the environment are high enough. When the product of shedding and acquisition is higher than the cost (i.e. *c* < *βγ* < *γ*), we get *T*_2_ > 1. Because *λ* is smaller than 1, by definition, *λ* is always smaller than *T*_2_. In this case, equilibrium 3 (*E*_*m*_ = 0) does not exist, and therefore the mutualist persists. That is, some of the individuals in the population still carry the mutualist despite highly unfaithful vertical transmission. To illustrate the effect of microbe acquisition from the environment on the steady states of the system we plot the thresholds *T*_1_ and *T*_2_ as functions of the acquisition rate *β* and *s* (Figure 3b and c). As *β* increases, *T*_2_ exceeds 1 (dashed line in panel b); extinction of the mutualist is prevented.

A positive fitness advantage allows the mutualist to persist in a population with some degree of leakiness *λ* even without acquisition or shedding. Both thresholds increase with the fitness benefit *s* of hosts with the mutualist as illustrated in Figure 3c. When the value of the fitness benefit *s* is small, the upper threshold is sensitive to changes in its value (Figure 3c). Hence, a slight increase in the fitness of the mutualist-carrying host allows the persistence of the mutualist to have a much higher tolerance for unfaithful vertical transmission.

However, as the fitness benefit *s* approaches infinity, *T*_2_ (Table 2) approaches 1. If the microbial shedding and acquisition are low compared with the fitness cost (*βγ* < *c*), an increase in host fitness increases the value of *T*_2_ while *T*_2_ is always smaller than 1. That is, an increase in host fitness allows the system to tolerate more leaky vertical transmission, but it will not prevent the eventual loss of the mutualist as leakiness increases (Figure 3c).

Overall, *T*_2_ is linear with respect to *βγ*/*c*, which can be viewed as a measure of the ‘strength’ of horizontal transmission, and it is the balance of these parameters that can sustain the presence of the mutualist even under extremely leaky conditions. In the following section we identify a generalisation of the *T*_2_ threshold that accounts for cultural factors and explore its dependence on other parameters.

### 3.2. Cultural factors can help the mutualist persist

In the extended model, we introduce the transmission of a cultural practice that affects the rate of acquisition of the mutualist. An example of the dynamics over time is shown in Figure S2 (Supplementary Material). We are interested in how cultural factors may affect the conditions under which the mutualist can enter the host population and persist. Thus, we investigate a boundary at which the mutualist is absent in the host population. From Equations 11 and 12, when *E*_*o*_ ≠ 0, we have *E*_*m*_ = *γ*(*M*_*x*_ + *M*_*y*_)/*c*. At the mutualist-free boundaries, 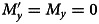 (Equation 7) and 
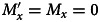
 (Equation 9); the mutualist proportion in the environment *E*_*m*_ then goes to zero. The resulting steady states and the conditions for stability are summarised in Table 3. The full mathematical analysis can be found in Supplementary Material 1.2.

**Table 3.** Non-negative equilibria and stability conditions at the mutualist-free boundaries. The mutualists are excluded at these equilibria (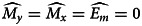)

	Equilibrium 1	Equilibrium 2
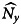	0	1 − *δ*/*k*
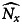	1	*δ*/*k*
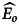	1	1
Stability conditions	*k* < *δ*	*k* > *δ*
	*β* < min(*c*/*γ*, 1)	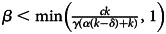
	*λ* > *T*_2_	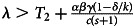

The cultural transmission model allows the adoption and abandonment of the cultural practice across generations. When the rate of abandonment is higher than the rate of adoption (*δ* > *k*), the system stabilises at equilibrium 1 where the cultural practice is excluded from the population (Table 3). A special case of this model where only one type of host (in the absence of the practice) exists at equilibrium (therefore no cultural factors) reduces to the basic model (Equations 1–4). Thus, the stability of equilibrium 1 depends on the upper threshold *T*_2_ of *λ* in the same manner as for the basic model (Table 2 and 3).

When the rate of adoption is higher than the rate of abandonment (*k* > *δ*), the system stabilises at equilibrium 2; a fraction of the population has the cultural practice. This proportion is determined by the ratio of the adoption and abandonment rate (Table 3). The stability of equilibrium 2 requires the leakiness of vertical transmission *λ* to be higher than a threshold 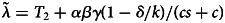. Therefore, increasing *α* (elevation in the rate of mutualist acquisition from the environment) and *k* (cultural transmission rate parameter) increases the value of *λ* above which the mutualist will go extinct (Table 3).

**Figure 4.**
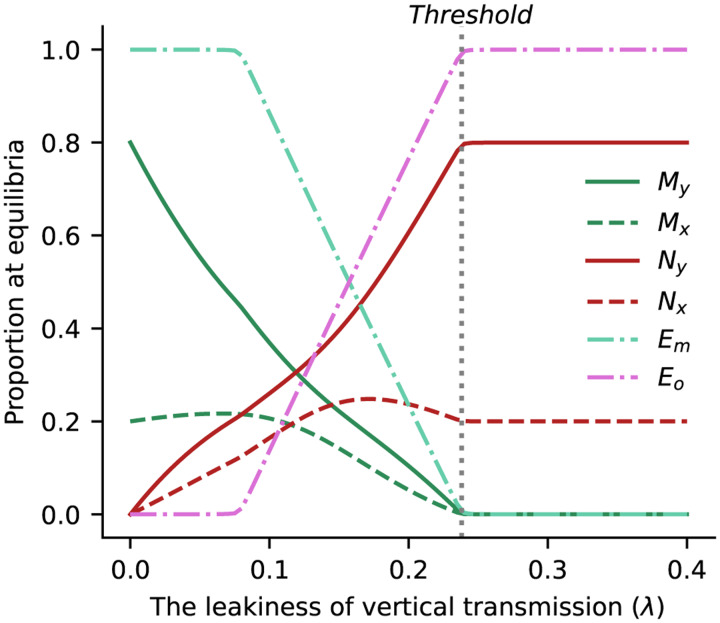
The proportion of microbiota types at equilibrium against *λ* (the leakiness of microbial vertical transmission) for the extended model with cultural practice. The curves show equilibria obtained numerically by solving Equations (7)–(12) using *γ* = 0.15, *λ* = 0.1, *β* = 0.1, *s* = 0.1, *c* = 0.1, *α* = *k* = 0.1 and *δ* = 0.02. An estimate of the equilibrium is obtained when the difference between two consecutive iterations is smaller than an error of 1 × 10^−5^. Increasing leakiness of vertical transmission reduces the proportion of the mutualist in the population and the environment. The black dotted line represents the threshold 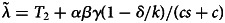.

Since *k* > *δ* is one of the conditions for stability, a stable equilibrium 2 ensures that the threshold leakiness is always greater than *T*_2_, as long as *α* is positive. Hence, transmission of a cultural factor that elevates microbial acquisition improves the ability of a mutualist to persist under leaky vertical transmission. On the other hand, a cultural practice that suppresses the acquisition of the mutualist (negative *α*) results in a threshold smaller than the basic model, which makes the mutualist more likely to go extinct owing to leaky vertical transmission. In Figure 4 we verify this threshold against the equilibria of the system computed with numerical solutions of Equations 7–12 across a range of *λ* values. As in the basic model, increasing the leakiness of vertical transmission *λ* decreases the proportion of mutualist carriers in the population (*M*_*y*_ and *M*_*x*_).

**Figure 5.**
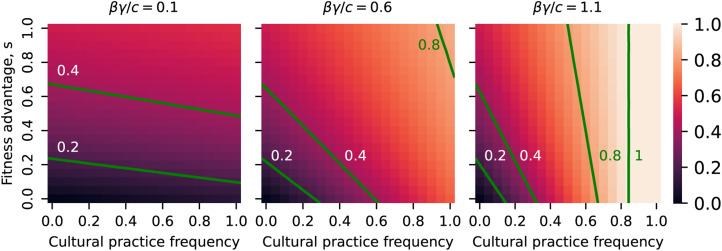
Heatmap of threshold leakiness 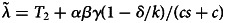 as a function of the equilibrium frequency of hosts with the cultural practice, 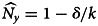 and benefit to the host *s* for three values of the strength of horizontal transmission *βγ*/*c*. The threshold leakiness 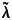 is the value of *λ* above which the mutualist will go extinct. The green lines are contours of the threshold at the values given in the labels. In all three heatmaps *α* = 0.1.

In Figure 5 we explore the behaviour of the threshold 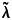 as a function of the equilibrium frequency of hosts with the cultural practice 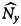, and the fitness benefit of the microbe to the host*s*. As the benefit to the host increases, the threshold above which the mutualist is excluded increases. As the equilibrium frequency of the cultural practice increases, the threshold increases linearly. The host–microbe association is able to tolerate greater leakiness in vertical transmission when the rate of adoption of the cultural trait *k* is greater than the rate of abandonment *δ*. The figure also shows that the threshold increases as a function of the compound parameter *βγ*/*c* which reflects the strength of horizontal transmission. Although not shown in the figure, the threshold leakiness also increases linearly with the elevated rate of acquisition from the environment owing to the cultural practice *α*.

The holobiont concept requires microbiota to be transmitted vertically with high fidelity (Rosenberg & Zilber-Rosenberg, 2016; Skillings, 2016). To study the dynamics of the mutualist in the context of holobionts, we investigate a special case of the model where vertical transmission is perfect. We solve the system (Equations 7–12) with *λ* = 0. The positive equilibria and corresponding conditions for stability are summarised in Table S1 (Supplementary Material). Under perfect vertical transmission, all individuals carry the mutualist because the two equilibria without the mutualist are both unstable, and some fraction of hosts have the cultural practice. The proportion of individuals with the cultural practice is an increasing function of the ratio of adoption and abandonment rates, *k*/*δ* (Table S1 in the Supplementary Material). The persistence of the mutualist under perfect vertical transmission is verified by our numerical solutions where microbiota without the mutualist *M*^−^ are eliminated under perfect vertical transmission *λ* = 0 (Figure 4). Therefore, perfect vertical transmission guarantees the presence of the mutualist at the individual level. However, as shown above, mutualists can persist in a population in the long run without this strict requirement.

The basic model (Equations 1–4) is a boundary of this extended model in which the individuals with the cultural practice are absent (*M*_*y*_ = *N*_*y*_ = 0). We can therefore use this model to consider a new cultural practice that appears in a population. A cultural practice can spread in the population if the rate of adoption is positive and greater than the rate of abandonment (Table 3 and Table S1 in the Supplementary Material). Interestingly, this criterion is independent of any properties of the mutualist, reflecting the fact that the cultural practice is itself selectively neutral.

## 4. Discussion

Disruption in the vertical transmission of the mutualist can affect beneficial host–microbe associations and change the structure of the gut microbiota permanently (Xiong et al., 2021). Here, we consider the dynamics of vertical and horizontal transmission to understand how mutualistic host–microbe associations can be maintained. The efficiency of vertical transmission strongly affects the microbe carrier frequency in the host population (Leftwich et al., 2020). Our results confirm this finding: the proportion of mutualist carriers decreases with imperfect vertical transmission and the mutualist eventually goes extinct if the transmission is too leaky. If horizontal transmission occurs (here, via an environmental reservoir) and allows the mutualist to enter the host population, then some degree of leakiness in vertical transmission is tolerated. Other work has shown that a selective advantage to the host can lead to higher frequencies of mutualist carriers in successive generations without a high parental contribution (Zeng et al., 2017). Likewise, we find that even if there is no environmental source and no horizontal transmission, as long as there is a fitness advantage (conferred to mutualist carriers), the mutualist can persist in the population despite imperfect vertical transmission.

The microbiota is transmitted through a mix of modes – a combination of vertical and horizontal transmission. This strategy allows symbionts to persist in a greater range of conditions, even when one form of transmission is unavailable or compromised (Ebert, 2013). Mutualistic relationships are believed to select for vertical transmission as a way to secure the advantage for the host and symbionts (Shapira, 2016). For example, a mutualist called *Bifidobacterium*, which is able to digest human milk, is transmitted from mother to infant during vaginal birth (Sela et al., 2008; Duranti et al., 2017). However, even when vertical transmission is interrupted, our analysis suggests that horizontal transmission can allow the mutualist to persist in some hosts. Therefore mixed-mode transmission can promote the persistence of the mutualist in a population, which in turn enables co-evolution between the host and the mutualist when vertical transmission is imperfect.

It is clear that vertical and horizontal transmission are both important mechanisms when considering host–mutualist associations and co-evolution. The holobiont theory assumes the stable inheritance of the microbiota (Skillings, 2016; Douglas & Werren, 2016), but in reality transmission is expected to be imperfect. It has been asserted that host–mutualist co-evolution is infeasible if the gut microbiota is not transmitted (to the next generation) with high fidelity (Douglas & Werren, 2016). We show, however, that horizontal transmission and a selective advantage to the host make it possible for the host–mutualist association to develop in a population without perfect vertical transmission. Under many conditions in our model with imperfect transmission, a fraction of the hosts continue to harbour the mutualist. Presumably, host–mutualist co-evolution can proceed without all individuals in a population carrying the mutualist. If a population can maintain a strong host–mutualist association, a host with its symbiont may arguably be considered an evolutionary unit.

Cultural evolution models have described the transmission of beneficial behaviours in a population (e.g. Boyd & Richerson, 2002). Further, in the context of microbiota establishment in non-human animals, social interaction increases exposure and susceptibility to symbiotic bacteria (Troyer, 1984) including mutualists (Lombardo, 2008; Archie & Tung, 2015) in a population. Here, we have examined a cultural practice that is selectively neutral to the host but facilitates access to the mutualist in the environment, which results in a higher rate of horizontal transmission. This implies that cultural factors can confer an indirect benefit to microbes by enabling their horizontal transmission. A variety of cultural practices may promote the establishment and persistence of mutualists in hosts. The high fibre diet of non-industrialised populations is associated with more diverse microbiomes and a much higher abundance of *Prevotella* compared with the microbiomes of industrialised populations (Schnorr et al., 2014; Clemente et al., 2015; De Filippo et al., 2010, 2017; Martínez et al., 2015). Infant-care practices such as pre-chewing facilitate maternal oral-to-infant microbial transmission in the Tsimane people of Bolivia (Sprockett et al., 2020). The consumption of fermented foods introduces and promotes mutualists in the gut (Kort et al., 2015; Kim et al., 2016). We have shown how cultural evolution can help to cement the host–microbe association, and reduce the impact of disrupted vertical transmission. If, however, a cultural practice decreases the rate of horizontal transmission it has the opposite effect of weakening the association. Our findings suggest that the complexity of human culture may have contributed to the wide variety of gut microbes as distinct microbiota patterns are found in different human communities (De Filippo et al., 2010; Rampelli et al., 2015).

In this article we have considered practices that alter horizontal but not vertical transmission; our model can be adapted in the future to address such effects. Another extension would be to consider the rates of horizontal and vertical transmission evolving as microbial rather than human traits. While we have focused on the ecological aspects of host–mutualist associations, the model can be extended to include microbe variation so that the symbiont can evolve and strengthen its association with (and benefit to) the host. This can be achieved by including multiple types of bacteria that affect the host fitness in different ways. Using the same framework, we can even study the evolution of a gut microbe that affects hosts in deleterious rather than beneficial ways.

## Research transparency and reproducibility

The data that support the findings of this study are openly available in [Gut-mutualists-can-persist-in-host-populations-despite-low-fidelity-of-vertical-transmission] at https://github.com/xiyanxiongnico/Gut-mutualists-can-persist-in-host-populations-despite-low-fidelity-of-vertical-transmission.git

## Supplementary material

For supplementary material accompanying this paper visit https://doi.org/10.1017/ehs.2022.38. click here to view supplementary material
