# Supplementary material for: Gut mutualists can persist in host populations despite low fidelity of vertical transmission
Source: Evol Hum Sci. 2022 Sep 2;4:e41. doi: 10.1017/ehs.2022.38 (PMC10426022; doi:10.1017/ehs.2022.38)
Supplement: Supplementary file 1 [file S2513843X2200038Xsup001.zip › S2513843X2200038Xsup001/Supplementary_material.pdf]

Supplementary material for *Gut mutualists can persist in host populations despite low fidelity of vertical transmission*

Xiyan Xiong, Sara L Loo, Mark M. Tanaka

School of Biotechnology and Biomolecular Sciences,
University of New South Wales,
Sydney, New South Wales 2052, Australia
and
Evolution & Ecology Research Centre, UNSW Sydney, Australia

1 Stability analysis

1.1 Basic model without cultural transmission

There are three feasible (non-zero) equilibria to the system (equations 1-4 in the main text). We investigate the stability conditions for each of these equilibria in the following sections.

1.1.1 Equilibrium 1

$$
\begin{aligned}
N &= \frac{\beta + \lambda - \sqrt{(\beta + \lambda + (\lambda - 1)s)^2 + 4\beta s} + \lambda s + s}{2s}, \\
M &= -\frac{\beta + \lambda - \sqrt{(\beta + \lambda + (\lambda - 1)s)^2 + 4\beta s} + \lambda s - s}{2s}, \\
E_m &= 1, \\
E_o &= 0.
\end{aligned}
$$

The jacobian matrix is

$$J = \begin{pmatrix} J_{11} & J_{12} & J_{13} & 0 \\ J_{21} & J_{22} & J_{23} & 0 \\ 0 & 0 & 0 & J_{34} \\ 0 & 0 & 0 & J_{44} \end{pmatrix}$$

where

$$J_{11} = \frac{2(s+1)(\beta+\lambda-1)\left(\beta+\lambda-\sqrt{(\beta+\lambda+(\lambda-1)s)^2+4\beta s}+\lambda s-s\right)}{s\left(\beta+\lambda-\sqrt{(\beta+\lambda+(\lambda-1)s)^2+4\beta s}+\lambda s-s-2\right)^2},$$

$$J_{12} = \frac{2(s+1)(\beta+\lambda-1)\left(\beta+\lambda-\sqrt{(\beta+\lambda+(\lambda-1)s)^2+4\beta s}+\lambda s+s\right)}{s\left(\beta+\lambda-\sqrt{(\beta+\lambda+(\lambda-1)s)^2+4\beta s}+\lambda s-s-2\right)^2},$$

$$J_{13} = \frac{\beta\left(\beta+\lambda-\sqrt{(\beta+\lambda+(\lambda-1)s)^2+4\beta s}+\lambda s+s\right)}{s\left(\beta+\lambda-\sqrt{(\beta+\lambda+(\lambda-1)s)^2+4\beta s}+\lambda s-s-2\right)},$$

$$J_{21} = -\frac{2(s+1)(\beta+\lambda-1)\left(\beta+\lambda-\sqrt{(\beta+\lambda+(\lambda-1)s)^2+4\beta s}+\lambda s-s\right)}{s\left(\beta+\lambda-\sqrt{(\beta+\lambda+(\lambda-1)s)^2+4\beta s}+\lambda s-s-2\right)^2},$$

$$J_{22} = -\frac{2(s+1)(\beta+\lambda-1)\left(\beta+\lambda-\sqrt{(\beta+\lambda+(\lambda-1)s)^2+4\beta s}+\lambda s+s\right)}{s\left(\beta+\lambda-\sqrt{(\beta+\lambda+(\lambda-1)s)^2+4\beta s}+\lambda s-s-2\right)^2},$$

$$J_{23} = -\frac{\beta\left(\beta+\lambda-\sqrt{(\beta+\lambda+(\lambda-1)s)^2+4\beta s}+\lambda s+s\right)}{s\left(\beta+\lambda-\sqrt{(\beta+\lambda+(\lambda-1)s)^2+4\beta s}+\lambda s-s-2\right)},$$

$$J_{34} = \frac{2s}{\beta\gamma+\gamma\lambda-\gamma\sqrt{(\beta+\lambda+(\lambda-1)s)^2+4\beta s}+\gamma\lambda s-\gamma s+2sc-2s},$$

$$J_{44} = \frac{1}{-\frac{\gamma\left(\beta+\lambda-\sqrt{(\beta+\lambda+(\lambda-1)s)^2+4\beta s}+\lambda s-s\right)}{2s}-c+1}.$$

Let v be the eigenvalue where $det(J - vI) = 0$. The eigenvalues are,

$$
\begin{aligned}
v_1 &= 0, \\
v_2 &= 0, \\
v_3 &= -\frac{2(s+1)(\beta+\lambda-1)}{D}, \\
v_4 &= \frac{1}{-\frac{\gamma\left(\beta+\lambda-\sqrt{(\beta+\lambda+(\lambda-1)s)^2+4\beta s}+\lambda s-s\right)}{2s} - c + 1},
\end{aligned}
$$

where

$$
\begin{aligned}
D =& \beta^2 + 2\beta\lambda - 2\beta + \lambda^2 - 2\lambda + \lambda^2 s^2 - 2\lambda s^2 + s^2 + 2\beta\lambda s - \\
& \lambda s\sqrt{(\beta+\lambda+(\lambda-1)s)^2+4\beta s} + s\sqrt{(\beta+\lambda+(\lambda-1)s)^2+4\beta s} - \\
& \beta\sqrt{(\beta+\lambda+(\lambda-1)s)^2+4\beta s} - \lambda\sqrt{(\beta+\lambda+(\lambda-1)s)^2+4\beta s} + \\
& 2\sqrt{(\beta+\lambda+(\lambda-1)s)^2+4\beta s} + 2\lambda^2 s - 4\lambda s + 2s + 2.
\end{aligned}
$$

Solving $v_3 < 1$ & $v_4 < 1$ we get $\lambda < \frac{\beta\gamma+cs}{c(s+1)} - \frac{\beta\gamma+cs}{\gamma(s+1)}$.

1.1.2 Equilibrium 2

$$
\begin{aligned}
N &= \frac{\lambda(s+1)c}{\beta\gamma+sc}, \\
M &= \frac{\beta\gamma-\lambda sc+sc-\lambda c}{\beta\gamma+sc}, \\
E_m &= \frac{\gamma(\beta\gamma+s(c-\lambda c)-\lambda c)}{c(\beta\gamma+sc)}, \\
E_o &= \frac{sc(\gamma(\lambda-1)+c)+\gamma(c(\beta+\lambda)-\beta\gamma)}{c(\beta\gamma+sc)}.
\end{aligned}
$$

The jacobian matrix is

$$
J = \begin{pmatrix}
J_{11} & \frac{\lambda(c-\beta\gamma)}{(\lambda-1)sc-\beta\gamma} & \frac{\beta\lambda c}{(\lambda-1)sc-\beta\gamma} & 0 \\
-\frac{(c-\beta\gamma)(-\beta\gamma+(\lambda-1)sc+\lambda c)}{(s+1)c((\lambda-1)sc-\beta\gamma)} & \frac{\lambda(c-\beta\gamma)}{\beta\gamma+s(c-\lambda c)} & \frac{\beta\lambda c}{\beta\gamma+s(c-\lambda c)} & 0 \\
0 & J_{32} & J_{33} & J_{34} \\
0 & J_{42} & J_{43} & J_{44},
\end{pmatrix}
$$

where

$$
\begin{aligned}
J_{11} &= \frac{(c-\beta\gamma)(-\beta\gamma+(\lambda-1)sc+\lambda c)}{(s+1)c((\lambda-1)sc-\beta\gamma)}, \\
J_{32} &= \frac{\gamma(sc(\gamma(\lambda-1)+c)+\gamma(c(\beta+\lambda)-\beta\gamma))}{c(\beta\gamma+sc)}, \\
J_{33} &= -\frac{(c-1)(sc(\gamma(\lambda-1)+c)+\gamma(c(\beta+\lambda)-\beta\gamma))}{c(\beta\gamma+sc)}, \\
J_{34} &= \frac{\gamma(-\beta\gamma+(\lambda-1)sc+\lambda c)}{c(\beta\gamma+sc)}, \\
J_{42} &= -\frac{\gamma(sc(\gamma(\lambda-1)+c)+\gamma(c(\beta+\lambda)-\beta\gamma))}{c(\beta\gamma+sc)}, \\
J_{43} &= \frac{(c-1)(sc(\gamma(\lambda-1)+c)+\gamma(c(\beta+\lambda)-\beta\gamma))}{c(\beta\gamma+sc)}, \\
J_{44} &= \frac{\gamma(\beta\gamma+s(c-\lambda c)-\lambda c)}{c(\beta\gamma+sc)}.
\end{aligned}
$$

Let v be the eigenvalues. The characteristic polynomial of the jacobian is

$$
\begin{aligned}
0 =& v^2((\lambda-1)s^3c^3v(\gamma\lambda-\gamma+c+v-1)+s^2c^2(c^2((\lambda-1)v+1)+c((\beta+1)\gamma(\lambda-1)+ \\
&(\lambda-1)v^2+v(\gamma(\beta(\lambda-2)+2\lambda^2-3\lambda+1)-\lambda+2)-1)+\beta\gamma(\gamma(\lambda-1)^2+ \\
&(\lambda-2)v^2-(2\gamma+1)(\lambda-1)v+1))+\gamma sc(c^2(\lambda+\beta(\lambda+(\lambda-2)v+2)+(\lambda-1)\lambda v) \\
&-\beta c(\gamma(-\beta\lambda+2\beta-2\lambda^2+\lambda+2)-(\lambda-2)v^2+v(\gamma(\beta+3\lambda-2)+ \\
&\lambda-4)+2)+\beta^2\gamma(-2\gamma(\lambda-1)-v^2+(\gamma-1)v+2))+\beta\gamma^2(c^2(\beta+\lambda) \\
&(-(-\lambda+v-1))-\beta c(\beta\gamma+2\gamma\lambda+\gamma+v^2-(\gamma+2)v+1)+\beta^2\gamma(\gamma-v+1))).
\end{aligned}
$$

Replace v with V where $V=v-1$, so that the condition for stability is $V<0$. Let the characteristic polynomial for V be $AV^2+BV+C=0$ where

$$
\begin{aligned}
A =& (s+1)c(\beta\gamma+sc)((\lambda-1)sc-\beta\gamma), \\
B =& (\lambda-1)s^3c^3(\gamma(\lambda-1)+c+1)+s^2c^2(\beta\gamma(-2\gamma(\lambda-1)+\lambda-3)+ \\
&(\lambda-1)c^2+c(\gamma(\beta(\lambda-2)+2\lambda^2-3\lambda+1)+\lambda))+\gamma sc(\beta^2(\gamma-3)\gamma+ \\
&c^2(\beta(\lambda-2)+(\lambda-1)\lambda)+\beta c(\lambda-\gamma(\beta+3\lambda-2)))-\beta\gamma^2\left(\beta^2\gamma+c^2(\beta+\lambda)-\beta\gamma c\right), \\
C =& (\beta\gamma+sc)((\lambda-1)s^2c^2(\gamma(\lambda-1)+c)+sc(-2\beta\gamma^2(\lambda-1)+ \\
&\lambda c^2+\gamma c(\beta(\lambda-2)+2(\lambda-1)\lambda))+\gamma(\beta^2\gamma^2+\lambda c^2(\beta+\lambda)-\beta\gamma c(\beta+2\lambda))).
\end{aligned}
$$

The sum of the roots equals $-B/A$ and the product of roots equals C/A. For both roots to be negative, we need $-B/A<0$ and $C/A>0$. From inspection,

we get $A > 0$ always. Therefore, the conditions for $V < 0$, and hence stability, are $B < 0$ and $C < 0$ simultaneously. The boundaries for our parameters are $s > 0$,$0 < \beta < 1$, $0 < \gamma < 1$, $0 < s_e < 1$ and $0 < \lambda < 1$. Solving $B < 0$ and $C < 0$ with those boundaries, we get

$$\frac{\beta\gamma + cs}{c(s+1)} - \frac{\beta\gamma + cs}{\gamma(s+1)} < \lambda < \frac{\beta\gamma + cs}{c(s+1)}.$$

1.1.3 Equilibrium 3

$$\begin{aligned} &N = 1, \\ &M = 0, \\ &E_m = 0, \\ &E_o = 1. \end{aligned}$$

The jacobian matrix is

$$J = \begin{pmatrix} 0 & (\lambda-1)(s+1) & -\beta & 0 \\ 0 & (\lambda-1)(-(s+1)) & \beta & 0 \\ 0 & \gamma & 1-c & 0 \\ 0 & -\gamma & c-1 & 0 \end{pmatrix}.$$

Let v be the eigenvalue where $det(J - vI) = 0$. We get $v^2((c+v-1)(\lambda+(\lambda-1)s+v-1)-\beta\gamma) = 0$.

The eigenvalues are

$$\begin{aligned} v_1 &= 0, \\ v_2 &= 0, \\ v_3 &= \frac{1}{2}\left(-\lambda - \sqrt{4\beta\gamma + \lambda^2 + (\lambda-1)^2 s^2 - 2(\lambda-1)s(c-\lambda) + c^2 - 2\lambda c} - \lambda s + s - c + 2\right), \\ v_4 &= \frac{1}{2}\left(-\lambda + \sqrt{4\beta\gamma + \lambda^2 + (\lambda-1)^2 s^2 - 2(\lambda-1)s(c-\lambda) + c^2 - 2\lambda c} - \lambda s + s - c + 2\right). \end{aligned}$$

Solving $v_3 < 1$ & $v_4 < 1$, we get $\lambda > \frac{\beta\gamma+sc}{sc+c}$

1.2 Model with cultural transmission

We explore the boundary of the model with cultural transmission (Equations 7-12 in the main text) where the mutualist is extinct. There are two feasible equilibria at this boundary. We investigate the stability conditions for each of these equilibria in the following sections.

1.2.1 Equilibrium 1

The jacobian matrix for $M_y = 0, N_y = 0, M_x = 0, N_x = 1, E_m = 0, E_o = 1$ is

$$J = \begin{pmatrix} -\delta - (\lambda - 1)(s+1) & 0 & 0 & 0 & 0 & 0 \\ \lambda(s+1) & -\delta + k + 1 & 0 & 0 & 0 & 0 \\ \delta & 0 & (\lambda-1)(-(s+1)) & 0 & \beta & 0 \\ -s-1 & \delta - k - 1 & (\lambda-1)(s+1) & 0 & -\beta & 0 \\ \gamma & 0 & \gamma & 0 & 1-c & 0 \\ -\gamma & 0 & -\gamma & 0 & c-1 & 0 \end{pmatrix}.$$

Let v be the eigenvalue where $det(J - vI) = 0$. We get, $v^2(-\delta+k-v+1)(-\delta-(\lambda-1)(s+1)-v)((c+v-1)(\lambda+(\lambda-1)s+v-1)-\beta\gamma) = 0$. Provided $s > 0 \wedge 0 < c < 1 \wedge 0 < \gamma < 1 \wedge 0 < \beta < 1 \wedge 0 < k < 1 \wedge 0 < \delta < 1 \wedge \alpha > 0 \wedge 0 < \lambda < 1$, the following conditions need to be satisfied for a stable equilibrium ($v < 1$),

$$\begin{aligned} &0 < s < -\frac{\lambda}{\lambda - 1}, \\ &0 < \gamma \leq c\lambda + c\lambda s - cs, \\ &0 < \beta < 1, \\ &0 < k < \delta. \end{aligned}$$

or

$$\begin{aligned} &0 < s < -\frac{\lambda}{\lambda - 1}, \\ &c\lambda + c\lambda s - cs < \gamma < 1, \\ &0 < \beta < \frac{c\lambda + c\lambda s - cs}{\gamma}, \\ &0 < k < \delta. \end{aligned}$$

1.2.2 Equilibrium 2

The jacobian matrix for $M_y = 0, N_y = \frac{k-\delta}{k}, M_x = 0, N_x = \frac{\delta}{k}, E_m = 0, E_o = 1$ is

$$J = \begin{pmatrix} -\delta - \lambda - \lambda s + s + 1 & 0 & 0 & 0 & \frac{(\alpha+1)\beta(k-\delta)}{k} & 0 \\ \frac{(s+1)(\delta+k(\lambda-1))}{k} & \frac{\delta}{k} & -\frac{(s+1)(k-\delta)}{k} & -\delta + \frac{\delta}{k} + k - 1 & -\frac{(\alpha+1)\beta(k-\delta)}{k} & 0 \\ \delta & 0 & (\lambda-1)(-(s+1)) & 0 & \frac{\beta\delta}{k} & 0 \\ -\frac{\delta(s+1)}{k} & -\frac{\delta}{k} & \frac{(s+1)(k\lambda-\delta)}{k} & \delta - \frac{\delta}{k} - k + 1 & -\frac{\beta\delta}{k} & 0 \\ \gamma & 0 & \gamma & 0 & 1-c & 0 \\ -\gamma & 0 & -\gamma & 0 & c-1 & 0 \end{pmatrix}.$$

Let v be the eigenvalue where $det(J - vI) = 0$. We get

$$v^2(-\delta + k + v - 1)(\beta\gamma\delta k(-\alpha\delta + (\alpha+1)k + \lambda + (\lambda-1)s + v - 1)$$
$$- k(\lambda + (\lambda-1)s + v - 1)(k(c + v - 1)(\delta + \lambda + (\lambda-1)s + v - 1) - (\alpha+1)\beta\gamma(k-\delta))) = 0$$

Provided $s > 0 \wedge 0 < c < 1 \wedge 0 < \gamma < 1 \wedge 0 < \beta < 1 \wedge 0 < k < 1 \wedge 0 < \delta < 1 \wedge \alpha > 0 \wedge 0 < \lambda < 1$, the following conditions need to be satisfied for a stable equilibrium ($v < 1$):

$$\begin{aligned} &0 < s < -\frac{\lambda}{\lambda-1}, \\ &0 < \gamma \leq c\lambda + c\lambda s - cs, \\ &0 < \beta < 1, \\ &\delta < k < 1, \\ &0 < \alpha < \frac{ck\lambda + ck\lambda s - cks - \beta\gamma k}{\beta\gamma k - \beta\gamma\delta} \end{aligned}$$

or

$$\begin{aligned} &0 < s < -\frac{\lambda}{\lambda-1}, \\ &c\lambda + c\lambda s - cs < \gamma < 1, \\ &0 < \beta < \frac{c\lambda + c\lambda s - cs}{\gamma}, \\ &\delta < k < 1, \\ &0 < \alpha < \frac{ck\lambda + ck\lambda s - cks - \beta\gamma k}{\beta\gamma k - \beta\gamma\delta}. \end{aligned}$$

2 Supplementary figures

This section includes the longitudinal dynamics of the basic model and the model with cultural transmission. Baseline parameter values ($\gamma = 0.1$, $\lambda = 0.1$, $\beta = 0.1$, $s = 0.1$, $c = 0.1$, $\alpha = 0.1$, $k = 0.1$ and $\delta = 0.02$) are chosen as an example to visualise the dynamics and illustrate the thresholds (Figure 3-5 in the main text).

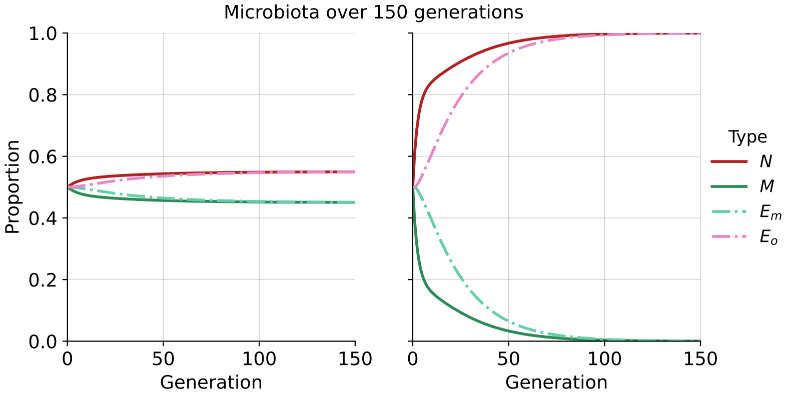

Figure S1: Proportions of microbiota types over time in the simple model using $\lambda = 0.1$ and $\lambda = 0.3$. Other parameters are set at baseline values. Initial conditions are set at 0.5 (equal proportion of host and microbiota types). The mutualists in the population (M_y and M_x) and the environment E_m are extinct under a more leaky vertical transmission ($\lambda = 0.3$).

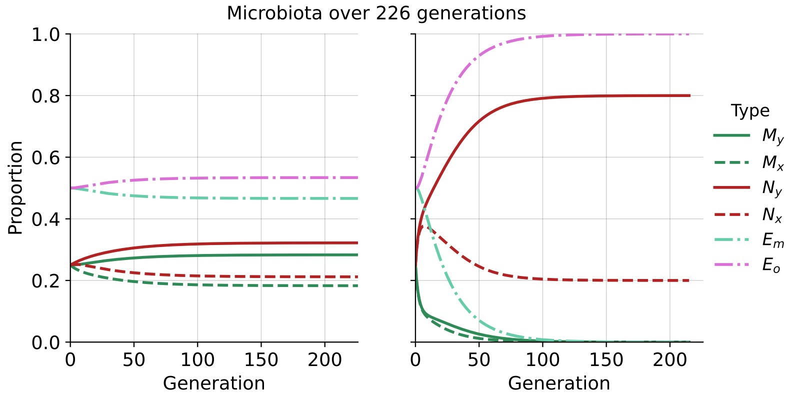

Figure S2: Proportions of microbiota types over time in the model with cultural transmission using $\lambda = 0.1$ and $\lambda = 0.3$. Other parameters are set at baseline values. Initial conditions are set at 0.25 (equal proportion of host and microbiota types). The mutualists in the population (M_y and M_x) and the environment E_m are extinct under a more leaky vertical transmission ($\lambda = 0.3$).

3 Supplementary table

This section include non-negative equilibria and stability conditions for the model with cultural transmission under perfect vertical transmission ($\lambda = 0$).

Table S1: Non-negative equilibria and the conditions for stability

	Equilibrium 1	Equilibrium 2	Equilibrium 3	Equilibrium 4
$\hat{M}_y$	0	0	0	$\frac{k-\delta}{k}$
$\hat{N}_y$	0	$\frac{k-\delta}{k}$	0	0
$\hat{M}_x$	0	0	1	$\frac{\delta}{k}$
$\hat{N}_x$	1	$\frac{\delta}{k}$	0	0
$\hat{E}_m$	0	0	$\frac{\gamma}{c}$	$\frac{\gamma}{c}$
$\hat{E}_o$	1	1	$1 - \frac{\gamma}{c}$	$1 - \frac{\gamma}{c}$
Stability conditions	Unstable	Unstable	$0 < k < \delta$<br>$\gamma < c < 1$	$\delta < k < 1$<br>$\gamma < c < 1$
